# Metabolome and Transcriptome Analysis of Liver and Oocytes of *Schizothorax o’connori* Raised in Captivity

**DOI:** 10.3389/fgene.2021.677066

**Published:** 2021-10-08

**Authors:** Jianshe Zhou, Wanliang Wang, Zhichao Li, Chi Zhang, Zhiyi Wan, Shuaijie Sun, Benhe Zeng, Ming Li, Guirong Sun

**Affiliations:** ^1^ College of Animal Science and Technology, Henan Agricultural University, Zhengzhou, China; ^2^ Institute of Fisheries Science, Tibet Academy of Agricultural and Animal Husbandry Sciences, Lhasa, China

**Keywords:** *Schizothorax o’connori*, metabolome, transcriptome, liver, oocyte, captivity

## Abstract

*Schizothorax o’connori* (*S. o’connori*) is a representative tetraploid species in the subfamily Schizothoracinae and an important endemic fish in the Qinghai-Tibet Plateau. However, the domestication of *S. o’connori* remains challenging due to the lack of basic research. Here, we investigated the effects of artificial feeding on the oocytes and liver of *S. o’connori* by comparing the histological, metabolomic, and transcriptomic data. Histological results showed that the oocytes and liver of captive-reared *S. o’connori* had abnormal cell morphology. After comparison with the self-built database, a total of 233 metabolites were annotated. In oocytes, a total of 37 differentially accumulated metabolites (DAMs) were detected and two pathways were significantly enriched. There were obvious differences in the metabolites related to ovarian development, including pregnenolone and arachidonic acid. In liver, a total of 70 DAMs were detected and five pathways were significantly enriched. Based on the transcriptomic data, a total of 159 differentially expressed genes (DEGs) were significantly related with cell growth and death pathway in oocytes, while a total of 2841 DEGs were significantly related with 102 pathways in liver. Comparing the metabolomic and transcriptomic data showed that there were three common significant enrichment pathways in liver, including biosynthesis of unsaturated fatty acids, starch and sucrose metabolism, and fatty acid biosynthesis. These results showed that special attention should be given to the composition and intake of fatty acids during the artificial breeding of *S. o’connori*. In addition, many of metabolite-gene pairs were related to adenosine 5′-diphosphate, adenosine monophosphate, and pregnenolone. In summary, these data provide an overview of global metabolic and transcriptomic resources and broaden our understanding of captive-reared *S. o’connori*.

## Introduction

*Schizothorax o’connori* (*S. o’connori*) is an important subfamily of endemic tetraploid fish in the main streams and tributaries of the Yarlung Tsangpo River (YLTR) on the Qinghai-Tibet Plateau (QTP) (Qi et al., 2012). It is also an excellent model organism for investigations of genome evolution and Quaternary climatic oscillations on the population structure of species endemic to the QTP (Guo et al., 2016; Xiao et al., 2020). For example, the study of the *S. o’connori* genome provided new insights into early re-diploidization and high-altitude adaptation of the endemic fish (Xiao et al., 2020). However, due to human activities and overfishing, the wild resources of *S. o’connori* have been declining in recent years. *S. o’connori* has been classified as a threatened species according to a recent investigation of fishery resources in the YLTR (Ma et al., 2020).

Domestication of *S. o’connori* is considered as an effective tool for the utilization and protection of the fish resources. Artificial breeding and reproduction of *S. o’connori* have been performed in recent years (Jing et al., 2011; Zhang, 2011). However, the domestication of *S. o’connori* remains many challenges, such as the fish may exhibit important reproductive dysfunctions when raised in captivity (Zhang, 2011). The gene expression profile of *S. o’connori* gonads is poorly understood. Multiple studies have shown that fish reproductive dysfunction comes from comprehensive stress under artificial breeding conditions, especially nutritional factors including fatty acids and vitamins, which play vital roles in egg quality and reproductive function (Izquierdo et al., 2001; Schreck et al., 2001; Mylonas et al., 2010; Rodríguez-Barreto et al., 2014). In addition, vitellogenin synthesized in the liver also plays an important role in the development of oocytes (Guzmán et al., 2008). In most teleost, the zona pellucida proteins are synthesized in the liver and subsequently transported to the growing oocytes (Modig et al., 2007). In the carp ovary, pregnenolone is synthesized from cholesterol which is synthesized in the liver (Mukherjee et al., 1991). Both liver and ovarian tissues play an important role in the reproductive process of various fish (Shirai et al., 2001; Hammond, 2011; Nagler et al., 2012; Qiao et al., 2019). However, effects of artificial feeding on the oocyte and liver of *S. o’connori* remain poorly understood.

Therefore, the objective of this study was to investigate the effects of artificial feeding on the oocytes and liver of *S. o’connori*. This was achieved by comparing the histology, metabolome, and transcriptome of the liver and oocytes in wild and captive-reared *S. o’connori*. By integrating metabolomic and transcriptomic data, we further identified potential differentially accumulated metabolites (DAMs) and the corresponding differentially expressed genes (DEGs) at the biochemical and molecular levels.

## Materials and Methods

### Experimental Animals and Animal Care

Before the spawning season, wild female *S. o’connori* individuals were captured in the YLTR in Tibet, and cultivated *S. o’connori* individuals were collected at the Institute of Fisheries Science, Tibet Academy of Agricultural and Animal Husbandry Sciences (Tibet, China). Cultivated *S. o’connori* individuals were fed with a formula feed containing 35% crude protein and 8% lipids twice a day at 3% body weight each time for more than 1 year. A total of four wild-type and four cultured female *S. o’connori* individuals were randomly selected for the experiment. All experiments with fish in this study were conducted in accordance with the guidelines on the care and use of animals for scientific purposes, established by the College of Animal Science and Technology of Henan Agricultural University, Zhengzhou, China (approval number: No. 20190521). After euthanizing, the liver and oocyte were collected. Part of the tissues was rapidly snap-frozen in liquid nitrogen and stored at −80°C, while the other part was fixed in Bouin’s fluid overnight at 4°C.

### Hematoxylin and Eosin Staining

The fixed samples were dehydrated in an alcohol gradient and xylene and then embedded in paraffin prior to being cut into 5-μm sections for HE staining. HE staining was performed as previously described (Feldman and Wolfe, 2014). Three discontinuous sections for each sample were selected and observed with a light microscope at 400X and 20X (Olympus, Japan).

### Metabolomics Analysis

For each tissue, approximately 50 mg of powdered sample was vortexed in 400 μl of cold mix (acetonitrile:methanol:water, 4:4:2) and then incubated for 60 min at −20°C. After centrifugation at 14,000 g for 20 min at 4°C, the supernatant was collected and dried in a vacuum desiccator. To end derivatization, the sample was vortexed in 100 μl of an acetonitrile:water mixture (1:1) for 1 min. After centrifugation at 14,000 g for 15 min at 4°C, 2 μl of supernatant was immediately analyzed using liquid chromatography electrospray ionization tandem mass spectrometry (LC-ESI-MS/MS).

Chromatographic separation was implemented on an ultra-high performance Liquid Chromatography (Agilent 1290 Infinity, Waldbronn, Germany). The column oven was maintained at 25°C. The flow rate was 0.3 ml/min. The injection volume for each sample was 2 μl. The mobile phase consisted of solvent A (water + 25 mM aqueous ammonia + 25 mM ammonia) and solvent B (acetonitrile). Gradient elution conditions were set as follows: 0–1 min, 95% phase B; 1–14 min, 95–65% B; 14–16 min, 65–40% B; 16–18 min, 40% B; 18–18.1 min, 40–95% B; 18.1–23 min, 95% B. The samples were continuously analyzed in a 4°C autosampler. The quality control samples were inserted into the sample queue to evaluate system stability throughout the experiment.

The metabolites were then analyzed using a high-resolution tandem mass spectrometry TripleTOF 6600 (AB SCIEX, United States) with an electronic spray ionization (ESI) source, operated in both positive and negative ion modes. The ESI source operation parameters were as follows: source temperature, 600°C; IonSpray Voltage Floating (ISVF), ±5500 V; ion source gas I (GSI), gas II (GSII), and curtain gas (CUR), 60, 60, and 30 psi, respectively. The time-of-flight (TOF) mass range was from 60 to 1000 Da, and the scan time was 0.2 s/spectra. The product ion scan range was from 25 to 1000 Da, and the product ion scan time was 0.06 s/spectra. The operation parameters of the high-sensitivity mode were as follows: declustering potential, ±60 V; collision energy, 35 ± 15 eV; excluding isotopes, within 4 Da; and candidate ions to monitor per cycle, 6.

The raw data were converted into mzXML format by ProteoWizard. Peak identification, filtration, and alignment were performed using XCMS program. The ion peaks with >50% missing values. The identification of each metabolite was performed according to the chemical formula generated from the accurate mass, with an accuracy of <25 ppm and MS/MS spectrogram matching. Metabolite identification was performed using a self-built database (Personalbio Technology Co., Ltd., Shanghai, China).

Principal component analysis (PCA), partial least square discriminant analysis (PLS-DA), and orthogonal partial least squares discriminant analysis (OPLS-DA) were performed after pattern recognition and pareto-scaling using SIMCA-P 14.1 (Umetrics, Umea, Sweden). The variable influence on projection (VIP) values were obtained from the OPLS-DA. Differentially accumulated metabolites (DAMs) were defined according to the following criteria: a VIP value > 1 and a q-value < 0.05. Clustering analyses of DAMs were applied based on Euclidean distance. Metabolic pathway analyses were performed using the Kyoto Encyclopedia of Genes and Genomes (KEGG) database (http://www.kegg.jp/). KEGG pathways with FDR < 0.05 were considered to be significantly enriched.

### Transcriptome Analysis

Total RNA was extracted using TRIzol reagent (Takara, Dalian, China) following the manufacturer’s instructions and treated with DNase I. RNA quantity and purity were assessed using the RNA Nano 6000 Assay Kit of the Bioanalyzer 2100 system (Agilent Technologies, CA, United States). Sequencing libraries were generated using the TruSeq RNA Sample Preparation Kit (Illumina, San Diego, CA, United States) following the manufacturer’s recommendations. The library was purified (AMPure XP system) and quantified using the Agilent high sensitivity DNA assay on a Bioanalyzer 2100 system (Agilent Technologies, CA, United States). The libraries were then sequenced on a HiSeq platform (Illumina, San Diego, CA, United States) by Shanghai Personal Biotechnology Cp. Ltd., and 150 bp paired reads were generated. The raw sequence files were deposited to the National Center for Biotechnology Information Sequence Read Archive with accession number SRP300881.

Raw reads were filtered to remove adaptor-containing reads, low quality reads (mean Q-value <20), and reads containing more than 5% ambiguous nucleotides using SOAPnuke (V2.0.7) (Chen et al., 2018). Clean reads were obtained and aligned to the *Schizothorax o’connori* genome (Xiao et al., 2020) using Bowtie2 v2.2.5 (Langmead and Salzberg, 2012). The gene expression level was estimated based on the unique mapped reads by the reads per kilobase million mapped reads (RPKM) method using RSEM v1.2.8 (Li and Dewey, 2011). Hierarchical clustering analyses were performed based on the Pearson correlation and average linkage method. Differentially expressed genes were identified by DESeq2 (v1.16) with a fold change (FC) > 2 and a false discovery rate (FDR) < 0.05 (Love et al., 2014). GO enrichment analysis was performed using Blast2GO software (Conesa et al., 2005). Pathway analysis of DEGs was performed using the KEGG PATHWAY database (http://www.kegg.jp) and Blast2GO (Conesa et al., 2005). GO and KEGG terms with FDR < 0.05 were considered to be significantly enriched. The Pathview package was used to visualize the results of association analysis of differential metabolites and related transcripts.

### Quantitative Real-Time PCR Analysis

qRT-PCR analysis was performed by using the ChamQ SYBR qPCR Master Mix (Vazyme Biotec, Nanjing, China) on a Light Cycler 480 system (Roche, Basel, Switzerland). A total of 10 DEGs related to fatty acid biosynthesis, biosynthesis of unsaturated fatty acids, and starch and sucrose metabolism, which are three significantly enriched pathways, are selected. Specific primers are listed in Supplementary Table S5. The expression levels of genes were normalized to the expression of the ACTB1 and calculated by the 2^−ΔΔCt^ method. All samples were carried out in three technical replicates on the same plate. Data were expressed as mean ± standard deviation. Pearson correlations were computed to evaluate the correlation between the RNA-seq and qRT-PCR data.

## Results

### Gross Morphology and Histology Analysis

**FIGURE 1:**
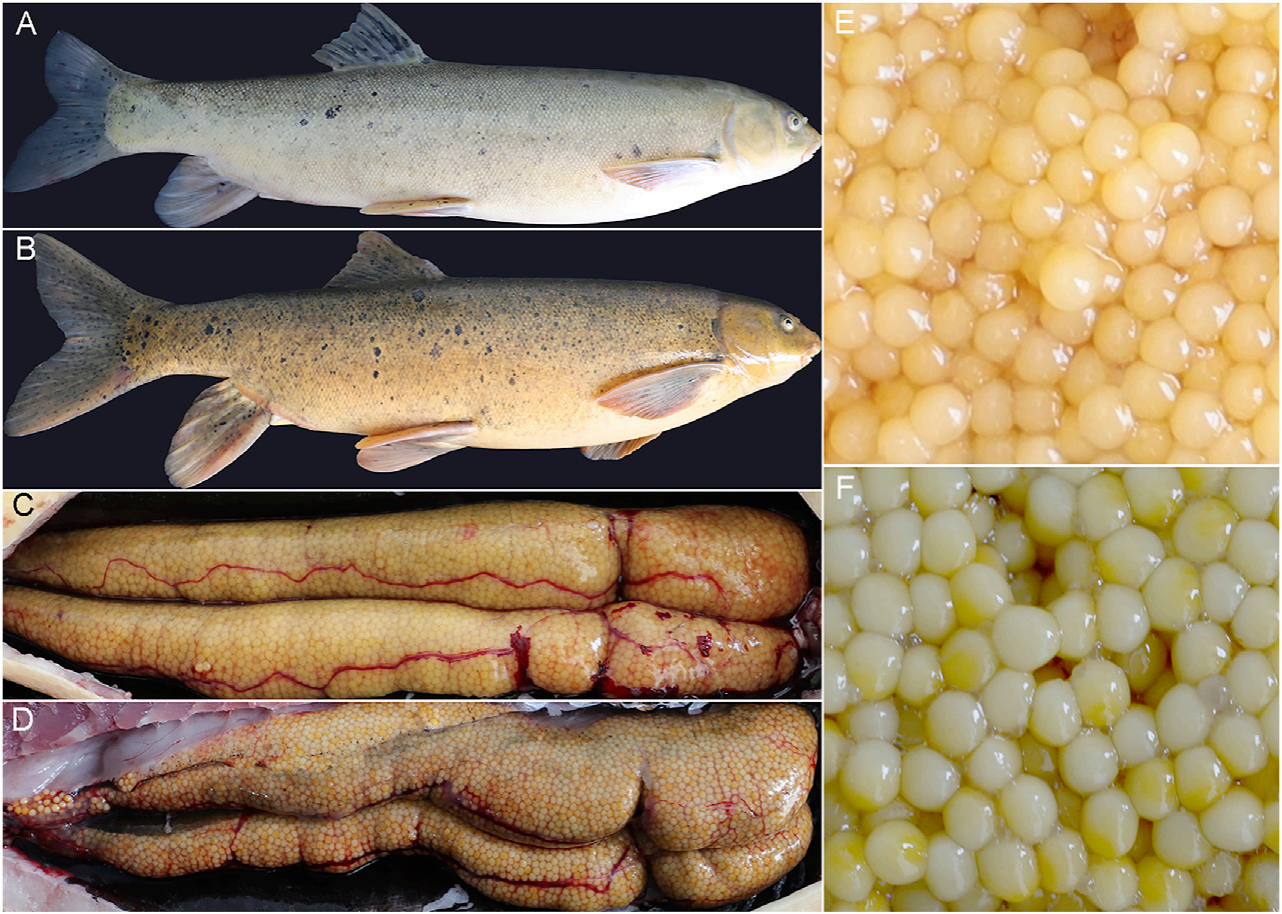
Gross morphology of *S. o’connori*, ovary, and ovum between wild and captive-reared group. **(A)** A photograph of wild *S. o’connori*. **(B)** A photograph of captive-reared *S. o’connori*. The ovary **(C)** and ovum **(E)** of wild *S. o’connori*. The ovary **(D)** and ovum **(F)** of captive-reared *S. o’connori*.

**FIGURE 2:**
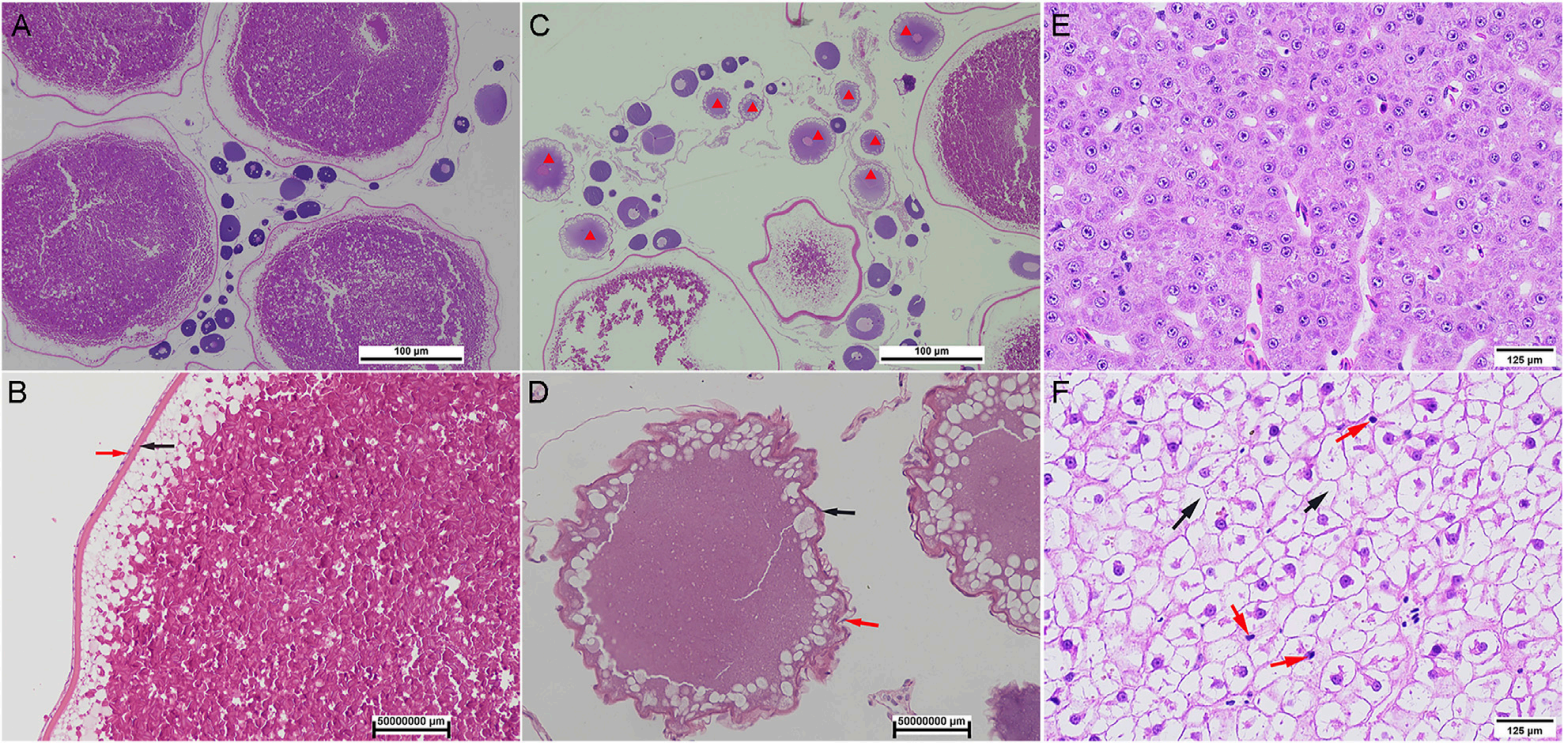
Histology of oocyte and liver in wild and captive-reared *S. o’connori*. **(A, B)** The oocytes at stages IV in ovaries of wild *S. o’connori*. **(C, D)** The oocytes at stages IV in ovaries of captive-reared *S. o’connori*. Red star: atresia oocytes; Black arrow: chorion; Red arrow: follicular cell layer. The liver in wild **(E)** and captive-reared **(F)** *S. o’connori*. Black arrow: liquefied cytoplasm.

Compared with wild *S. o’connori*, captive-reared *S. o’connori* has a yellow body color and nonuniformly sized whitish ova (Figure 1). Histological examination further showed that compared with wild *S. o’connori*, there were many atresia oocytes in the ovaries of captive-reared *S. o’connori*. The chorion and follicular cell layer also showed irregular shrinkage and folding (Figures 2A–D). The liver cells were evenly distributed in wild *S. o’connori*, while the nucleus was absent or pushed to the edge of the cell (Figures 2E,F, red arrows), and the cytoplasm was liquefied in the liver of captive-reared *S. o’connori* (Figures 2E,F, black arrows).

### Metabolic Profile Analysis

Metabolites were further analyzed using a liquid chromatography electrospray ionization tandem mass spectrometry (LC-ESI-MS/MS) system. The retention time and peak intensities of the quality control (QC) samples were reproducible, indicating that LC-ESI-MS/MS was reliable (Supplementary Figure S1). After raw data were preprocessed, 5,129 metabolite peaks (2,671 peaks in ESI positive mode and 2,458 peaks in ESI negative mode) were obtained for further analysis. The results of PCA, PLS-DA, and OPLSDA showed that the wild and captive-reared groups were clearly different in both the liver and the oocytes, reflecting significant metabolic differences among the different groups (Table 1, Supplementary Figures S2, S3). All samples fell within the 95% confidence interval.

**TABLE 1:** Multivariate analysis of metabolites in wild and captive-reared *S. o’connori*.

Multivariate analysis	Group	Positive ion model						Negative ion model					
		A	R^2^X	R^2^Y	Q^2^	R^2^ intercept	Q^2^ intercept	A	R^2^X	R^2^Y	Q^2^	R^2^ intercept	Q^2^ intercept
PCA	QC	3	0.507		0.219			3	0.532		0.243		
	OD vs OW	2	0.503		0.0346			2	0.529		0.0701		
	LD vs LW	2	0.495		−0.043			2	0.542		0.0148		
PLS-DA	OD vs OW	2	0.5	0.998	0.852			2	0.521	0.997	0.924		
	LD vs LW	2	0.469	0.999	0.907			2	0.533	0.999	0.942		
OPLS-DA	OD vs OW	1 + 3 + 0	0.714	1	0.943	1	0.614	1 + 4 + 0	0.82	1	0.958	1	0.787
	LD vs LW	1 + 3 + 0	0.725	1	0.921	1	0.633	1 + 3 + 0	0.752	1	0.961	1	0.53

PCA, principal component analysis; PLS-DA, partial least squares-discriminant analysis; OPLS-DA, orthogonal partial least squares-discriminant analysis; QC, quality control; LW, liver from wild *S. o’connori*; LD, liver from domestic *S. o’connori*; OW, oocyte from wild *S. o’connori*; OD, oocyte from domestic *S. o’connori*.

**FIGURE 3:**
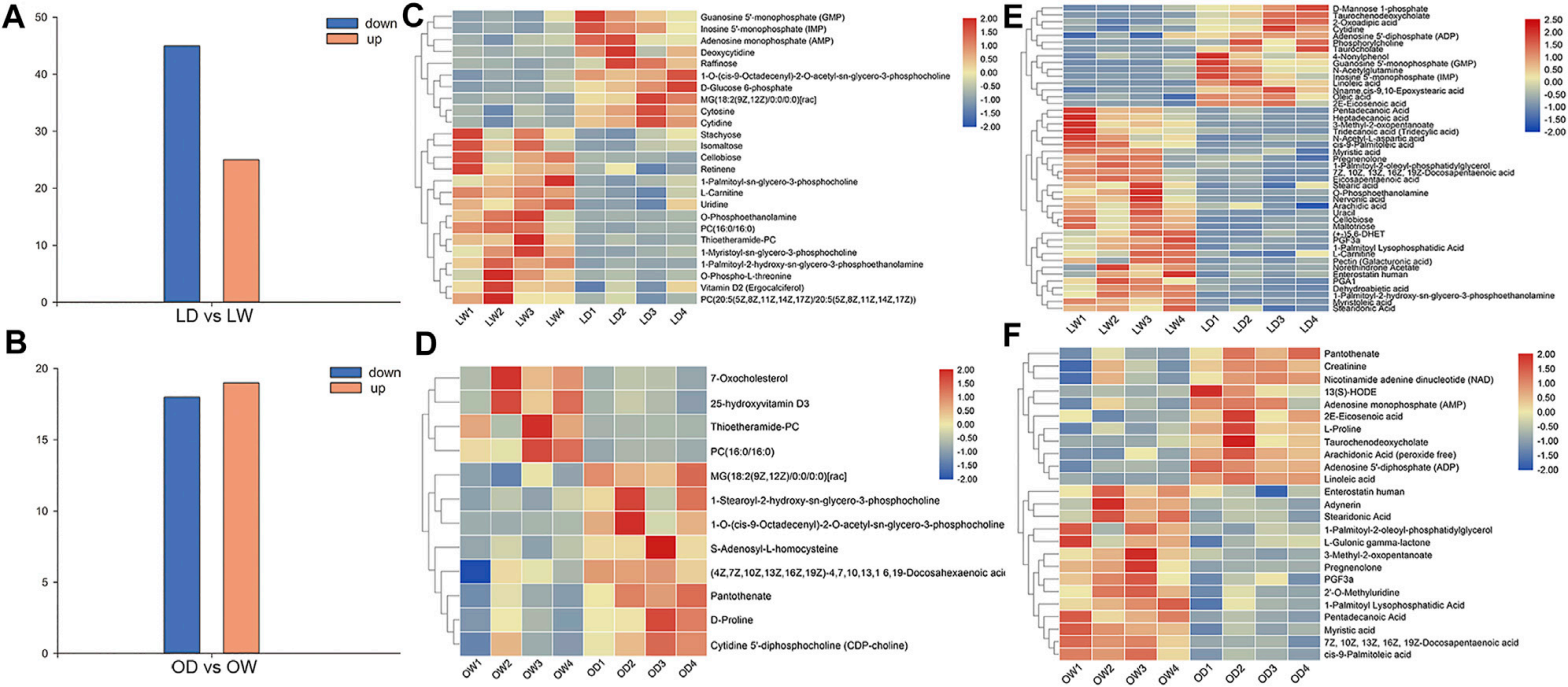
Differentially accumulated metabolites (DAMs) between the wild and captive-reared *S. o’connori* in both liver and oocyte. Number of DAMs in liver **(A)** and oocyte **(B)**. Hierarchical clustering analysis of DAMs using both positive **(C, D)** and negative **(E, F)** ionization mode in liver **(C, E)** and oocyte **(D, F)**. LW, liver from wild *S. o’connori*; LD, liver from domestic *S. o’connori*; OW, oocyte from wild *S. o’connori*; OD, oocyte from domestic *S. o’connori*.

After comparison with the self-built database, a total of 233 metabolites were annotated. There were 12 and 25 differentially accumulated metabolites (DAMs) between oocyte from domestic *S. o’connori* (OD) and oocyte from wild *S. o’connori* (OW) in the ESI+ and ESI− modes, respectively (Supplementary Table S1). However, there were 25 and 45 DAMs between liver from domestic *S. o’connori* (LD) and liver from domestic *S. o’connori* (LW) in the ESI+ and ESI− modes, respectively (Supplementary Table S1). Of the 37 DAMs identified between OD and OW, 18 (48.65%) and 19 (51.35%) metabolites were downregulated and upregulated, respectively. These DAMs included many metabolites related to ovarian development, such as pregnenolone, prostaglandin F3a (PGF3a), arachidonic acid (ARA), adenosine 5′- diphosphate (ADP), and adenosine monophosphate (AMP). Of the 70 DAMs in LD compared to LW, 45 (64.29%) and 25 (35.71%) metabolites were downregulated and upregulated, respectively (Figures 3A,B). A hierarchical clustering analysis was further performed to assess the DAMs accumulation patterns (Figures 3C–F).

According to the previous KEGG analysis, only two pathways were significantly enriched between the OD and OW group. These included arginine and proline metabolism and linoleic acid metabolism. There were five significantly enriched pathways between LD and LW, including the biosynthesis of unsaturated fatty acids, pyrimidine metabolism, ABC transporters, starch and sucrose metabolism, and fatty acid biosynthesis (Supplementary Table S2).

### General Analysis of Gene Expression Profiles Between Wild and Captive-Reared *S. o’connori*

**FIGURE 4:**
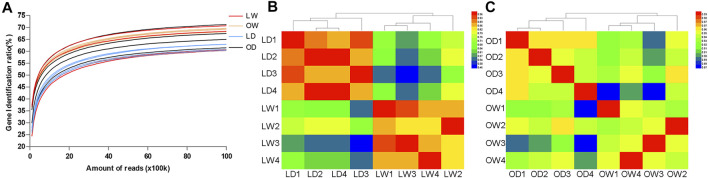
**(A)** Saturation curves analysis of all the biological samples. **(B)** Correlation analysis and hierarchical clustering of all the biological samples. LW, liver from wild *S. o’connori*; LD, liver from domestic *S. o’connori*; OW, oocyte from wild *S. o’connori*; OD, oocyte from domestic *S. o’connori*.

To provide an overview of the system-wide changes in gene expression during artificial breeding, transcriptomic profiles were investigated using RNA-Seq. After data filtering, a total of 658.68 M clean reads were acquired, ranging from 39.4 to 45.1 M clean reads per sample. Among these, more than 88.56 and 72.16% were mapped to the *S. o’connori* genome and the exonic regions for each sample, respectively. However, the average percentage of uniquely mapped reads of exonic regions was only 21.10%, indicating large number of duplicated genes (Table 2). Saturation curve analysis showed that the results of gene quantification were reliable (Figure 4A). Correlation analysis and cluster analysis were performed to gain insight into the relationships among different samples. Almost all the biological duplicates were also clustered together, confirming the relevance of the samples (Figures 4B,C).

**TABLE 2:** Summary of RNA-seq reads mapping to reference genome.

Samples	Clean reads	Mapping to genome rate (%)	Mapping to exon rate (%)	Uniq-mapping exon rate (%)
LD1	39.85	89.81	77.04	18.81
LD2	41.01	88.56	73.32	19.95
LD3	41.16	89.31	75.26	20.34
LD4	41.20	89.06	72.16	20.04
LW1	41.41	92.26	78.28	19.73
LW2	45.07	90.87	75.85	20.47
LW3	41.53	92.86	80.00	19.68
LW4	43.00	91.51	77.58	18.44
OD1	40.48	92.09	80.80	22.33
OD2	40.58	92.12	80.81	22.70
OD3	40.22	92.01	80.68	21.92
OD4	40.50	92.38	80.49	22.20
OW1	41.03	93.04	82.48	22.19
OW2	39.40	93.11	81.81	22.64
OW3	41.01	93.40	81.81	23.31
OW4	41.23	93.09	81.79	22.88

LW, liver from wild *S. o’connori*; LD, liver from domestic *S. o’connori*; OW, oocyte from wild *S. o’connori*; OD, oocyte from domestic *S. o’connori*.

**FIGURE 5:**
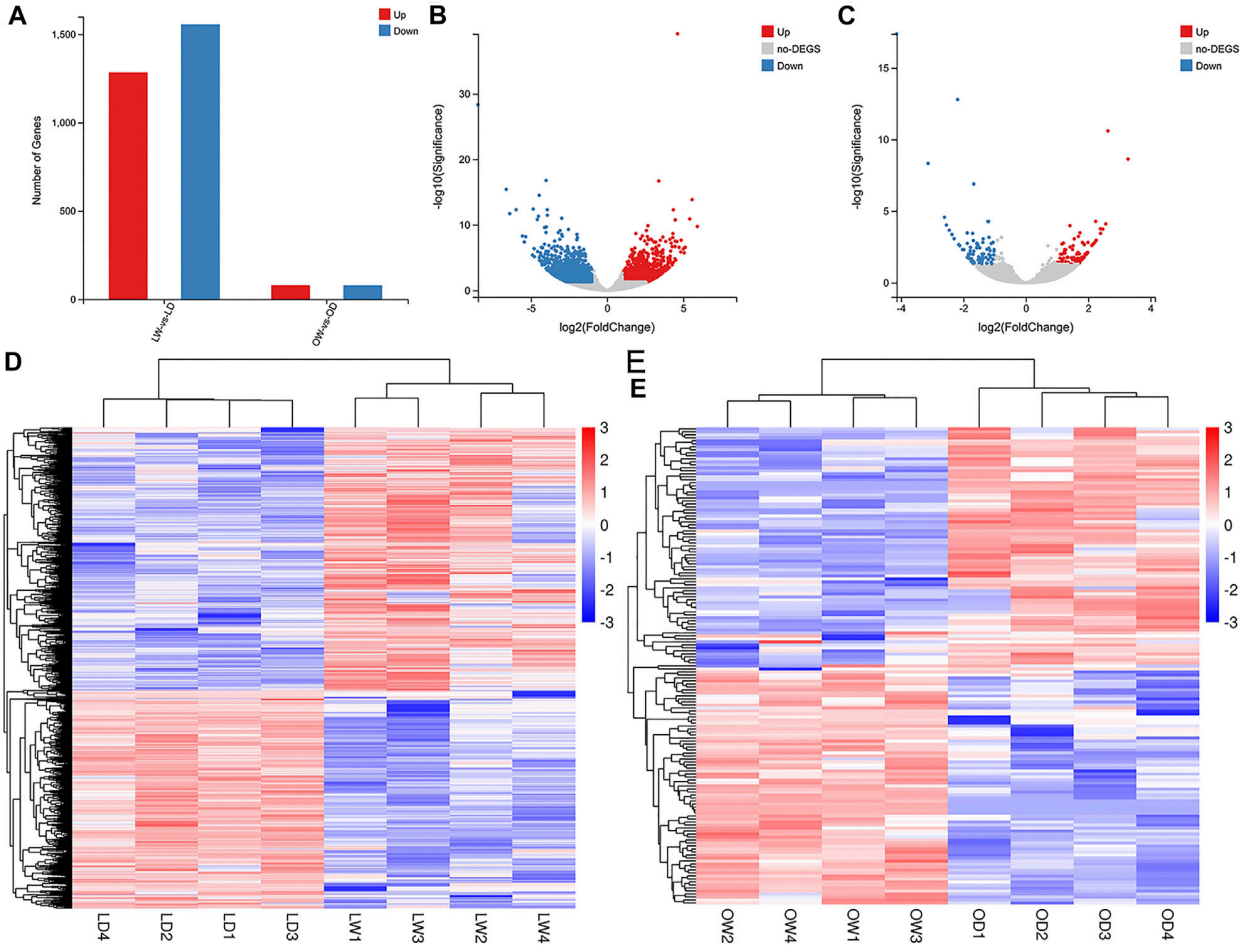
Analysis of differently expressed genes (DEGs) between the wild and captive-reared *S. o’connori*. **(A)** Number of DEGs in liver and oocyte between the wild and captive-reared *S. o’connori*. Volcano plot showing the DEGs in liver **(B)** and oocyte **(C)**. Hierarchical clustering of DEGs in liver **(D)** and oocyte **(E)**. Each row represents a gene and each column represents a sample. Each cell in the matrix corresponds to an expression level, with blue for under-expression, red for overexpression, and white for gene expression close to the median (see color scale). LW, liver from wild *S. o’connori*; LD, liver from domestic *S. o’connori*; OW, oocyte from wild *S. o’connori*; OD, oocyte from domestic *S. o’connori*.

A total of 2,841 DEGs (1,285 upregulated and 1,556 downregulated) were found in LD compared with LW, and 159 DEGs (80 upregulated and 79 downregulated) were found in OD compared with OW (Figures 5A–C, Supplementary Table S3). All DEGs between the wild and captive-reared *S. o’connori* were classified using hierarchical clustering (Figures 5D,E).

**FIGURE 6:**
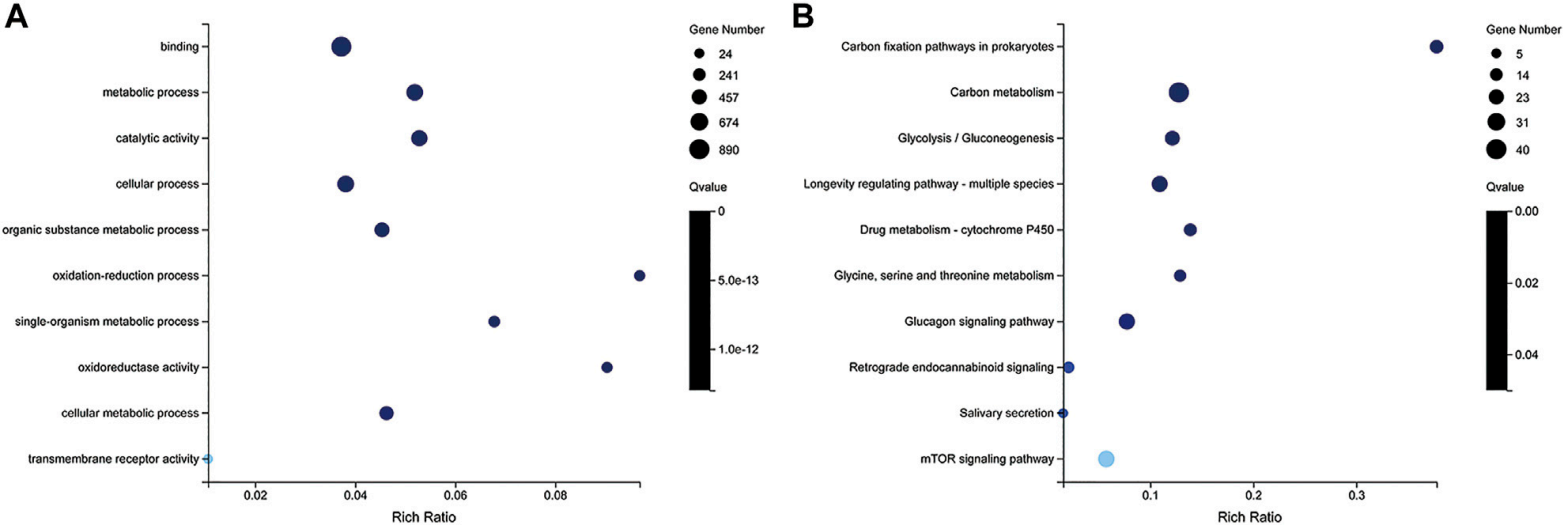
GO **(A)** and KEGG **(B)** enrichment analysis of differently expressed genes (DEGs) between LD and LW. The y-axis represents the enrichment pathway, while the x-axis represents the enrichment factor. The dot sizes represent the number of DEGs. LW, liver from wild *S. o’connori*; LD, liver from domestic *S. o’connori*.

To further understand the functions of these DEGs, GO and KEGG analyses were performed. Between LD and LW, a total of 449 GO terms and 102 pathways were significantly enriched, respectively (Supplementary Table S4). The top 10 significantly enriched GO terms and pathways are shown in Figure 6, including carbon fixation pathways in prokaryotes, PPAR signaling pathway neuroactive ligand-receptor interaction, propanoate metabolism, and carbon metabolism. However, only one GO term and one pathway (cell growth and death) were significantly enriched between OD and OW.

**FIGURE 7:**
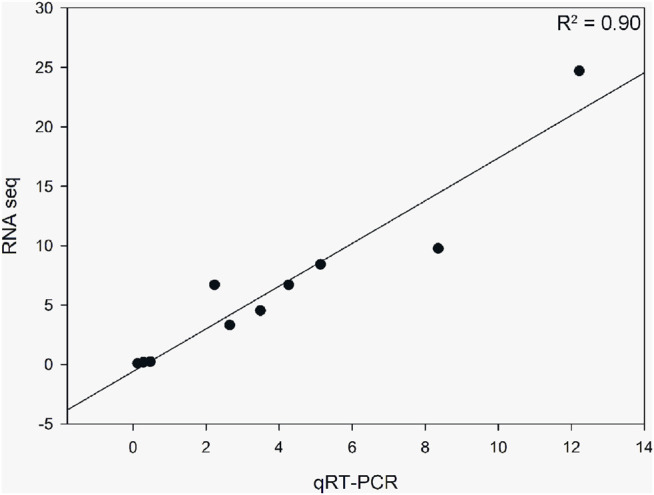
Correlation analysis of 10 differentially expressed genes obtained from RNA-seq and qRT-PCR. The qRT-PCR values were normalized relative to the expression levels of ACTB1 in the same sample. Data are expressed as the mean of three biological replicates.

Based on the DEG pathway enrichment results, qRT-PCR of 10 DEGs (*ACACA*, *ELOV6*, *FADS2*, *FAS*, *PGM1*, *SCD5*, *LYAG*, *G6PC3*, *HXK2*, and *ACBG1*) related to fatty acid biosynthesis, biosynthesis of unsaturated fatty acids, and starch and sucrose metabolism was performed to validate the differential gene expression obtained by RNA-seq. The results showed a strong correlation between the RNA-seq and qRT-PCR data (R^2^ = 0.90), indicating the reliability of the RNA-seq data (Figure 7).

### Association Analysis of the Metabolomic and Transcriptomic Data

**FIGURE 8:**
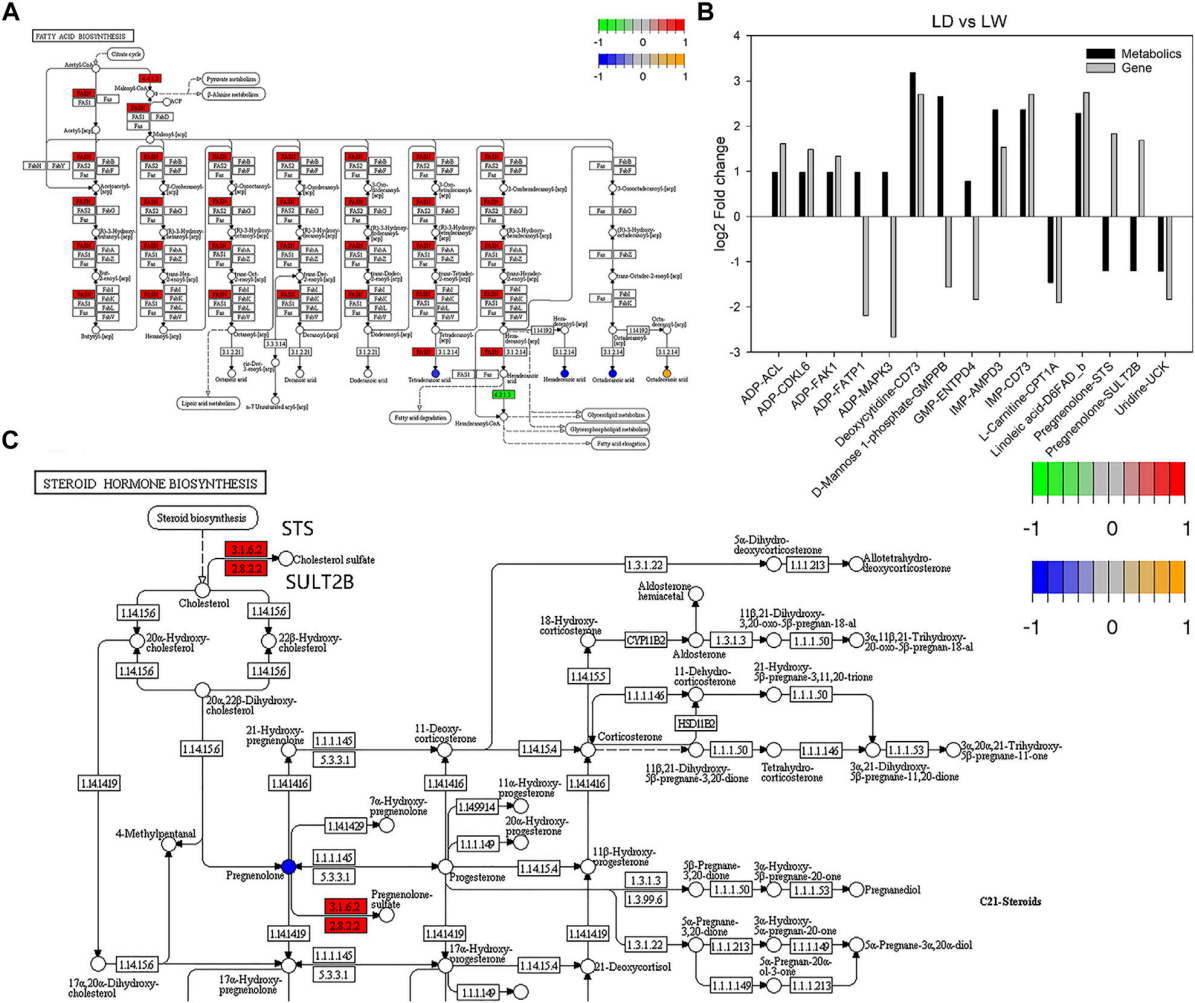
Integrated analysis of the accumulated metabolites (DAMs) and differently expressed genes (DEGs). **(A)** Fatty acid synthesis pathway. **(B)** Top 15 metabolite-gene pairs between LD and LW. **(C)** Part of steroid hormone biosynthesis pathway. The round nodes are metabolites, and the square nodes are the enzymes corresponding to the transcripts. The expression fold change of metabolites from low to high is represented by blue to yellow, and the expression fold change of transcripts from low to high is represented by green to red. ADP, adenosine monophosphate; GMP, guanosine 5′-monophosphate; IMP, inosine 5′-monophosphate; *ACL*, ATP-citrate synthase; *AMPD3*, AMP deaminase 3-like; *CDKL6*, cyclin-dependent kinase 6-like; *CPT1A*, carnitine O-palmitoyltransferase 1, liver isoform; *D6FAD_b*, delta-6 fatty acyl desaturase; *ENTPD4*, ectonucleoside triphosphate diphosphohydrolase 4-like; *FAK1*, focal adhesion kinase 1; *FATP1*, long-chain fatty acid transport protein 1; *GMPPB*, mannose-1-phosphate guanyltransferase beta; *MAPK3*, mitogen-activated protein kinase-activated protein kinase 3; *STS*, steryl-sulfatase; *SULT2B*, alcohol sulfotransferase; *UCK*, uridine-cytidine kinase; EC:2.8.2.2, SULT2B; EC:3.1.6.2, STS.

Comparing the metabolomic and transcriptomic data showed that there were three common significant enrichment pathways between LD and LW, including biosynthesis of unsaturated fatty acids, starch and sucrose metabolism, and fatty acid biosynthesis. The DAMs and DEGs related to the fatty acid synthesis pathway are shown in Figure 8A. However, no common pathway was detected between OD and OW. Notably, two DEGs (*CDC25B* and *FZR1*) in the progesterone-mediated oocyte maturation pathway were enriched, although the entire pathway was not significantly enriched. We further analyzed the metabolite-gene pairs using pathway-based integration method. Detailed information about the metabolite-gene pairs is shown in Supplementary Table S6. Top 15 metabolite-gene pairs between LD and LW are shown in Figure 8B. Of these, two metabolite-gene pairs including pregnenolone-*STS* and pregnenolone-*SULT2B* were negatively correlated (Figure 8B). These two metabolite-gene pairs are related to steroid hormone biosynthesis pathway (Figure 8C). In addition, the levels of pregnenolone and *LDLR* were all decreased in captive-reared *S. o’connori*.

## Discussion

*S. o’connori* is a representative tetraploid species in the subfamily Schizothoracinae and an important endemic fish in the QTP (Qi et al., 2012). However, its domestication remains challenging due to the lack of basic research. We report here the first histological, metabolomic, and transcriptomic analysis of liver and oocytes in wild and captive-reared *S. o’connori*.

Histological results showed that there were many atresia oocytes in captive-reared *S. o’connori*. Metabolomics data further revealed obvious differences in the metabolites related to ovarian development between the oocytes of wild and captive-reared *S. o’connori*, including pregnenolone, ARA, ADP, AMP, and PGF3a. Pregnenolone is an endogenous steroid and precursor/metabolic intermediate in the biosynthesis of most steroid hormones, such as estrogens, progestogens, glucocorticoids, androgens, and mineralocorticoids (Vallée, 2016). Studies in zebrafish, *Scomber japonicus*, and *Pimephales promelas* reported that pregnenolone plays an important role in the steroidogenesis pathway of the oocyte (Matsuyama et al., 2005; Petersen et al., 2015; Shang et al., 2019). ARA is a polyunsaturated fatty acid present in phospholipids and has an important role in reproduction (Tocher, 2003; Xu et al., 2016; Norberg et al., 2017). Oocyte maturation is inhibited by ADP and AMP (Miller and Behrman, 1986). Here, our data showed that the levels of pregnenolone and PGF3a decreased significantly in captive-reared *S. o’connori*, while the levels of ARA, ADP, and AMP increased significantly. Transcriptomic analysis also showed that the expression levels of *CDC25B* and *FZR1*, two genes related to progesterone-mediated oocyte maturation, were significantly different.

In addition, many studies have shown that the liver also plays important roles in the reproductive processes of various fish (Shirai et al., 2001; Nagler et al., 2012; Qiao et al., 2019). Here, HE staining showed that the cell morphology in the liver of captive-reared *S. o’connori* was abnormal. Based on the transcriptomic data between the livers of wild and captive-reared *S. o’connori*, a total of 2841 DEGs were significantly enriched in 102 pathways. Integrating the metabolomic and transcriptomic data revealed three common and significantly enriched pathways between the liver of wild and captive-reared *S. o’connori*, including biosynthesis of unsaturated fatty acids, starch and sucrose metabolism, and fatty acid biosynthesis. Fatty acids are not only the major source of metabolic energy in fish for growth, but are also very important in the reproduction of several fish species (Tocher, 2003; Tocher, 2010; Rodríguez-Barreto et al., 2014; Zupa et al., 2017). A report in gilthead seabream showed that dietary fatty acids were mostly incorporated into the ovary and liver (Harel et al., 1994). The biochemical composition of organs involved in reproduction is highly sensitive to the diet, which affects egg and larval quality (Harel et al., 1994). In Atlantic cod, ARA levels in both liver and ovary were correlated to dietary ARA levels (Norberg et al., 2017). In Asian seabass, the pathway of biosynthesis of unsaturated fatty acids was also significantly different when the fish were fed with different pelleted feeds (Ngoh et al., 2015).

The conversion of food habits is one of the greatest stresses for fish from wild to captive breeding. Nutrition is ultimately important in affecting egg quality and reproductive function (Schreck et al., 2001). Here, wild female *S. o’connori* individuals were captured in the YLTR before the spawning season. Diatom algae are the most important food for wild *S. o’connori* in the YLTR (Xie et al., 1994). Diatom algae store a high amount of fatty acids (Li et al., 2014; Tiwari et al., 2021). Cultivated *S. o’connori* individuals were fed with a formula feed containing 35% crude protein and 8% lipids for more than 1 year. Our results showed that special attention should be given to the composition and intake of fatty acids during the artificial breeding of *S. o’connori*.

Integrating the metabolomic and transcriptomic data also revealed kinds of metabolite-gene pairs. These metabolite-gene pairs contain 23 DAMs and 154 DEGs. Many of them were related to ADP, AMP, and pregnenolone. Here, we focused on the pregnenolone related metabolite-gene pairs, such as pregnenolone-*STS*, pregnenolone-*SULT2B*, and pregnenolone-*LDLR*. These metabolite-gene pairs are related to steroid hormone biosynthesis pathway which is a key pathway regulating ovarian development in fish species (Xu et al., 2016). STS and SULT2B catalyze the sulfonation of cholesterol and pregnenolone and have an important role in regulating the synthesis of estrogenic steroids (Ji et al., 2007; Purohit et al., 2011; Abunnaja et al., 2018). In *Coilia nasus*, *SULT2B1* expression is significantly increased during ovary development (Xu et al., 2016). Our results revealed that the levels of pregnenolone and *STS* or *SULT2B* were negatively correlated in captive-reared *S. o’connori*. All steroid hormones including pregnenolone are synthesized from cholesterol, while cholesterol is endocytosed into the cell by LDLR (Go and Mani, 2012). Our results also showed the levels of pregnenolone and *LDLR* were all decreased in captive-reared *S. o’connori*.

In conclusion, the present work is the first comparative metabolomic and transcriptomic study of liver and oocyte between the wild and captive-reared *S. o’connori*. These data provide an overview of the global metabolic and transcriptomic changes in the liver and oocytes during artificial feeding. In oocytes, some DAMs and DEGs were annotated as related to progesterone-mediated oocyte maturation. In the liver, the DAMs and DEGs were significantly enriched in the pathways of biosynthesis of unsaturated fatty acids, starch and sucrose metabolism, and fatty acid biosynthesis, which indicated that these may be considered key metabolic pathways during artificial feeding. These results broaden our understanding of reproductive physiology in captive-reared *S. o’connori*.

## Data Availability

The data presented in this study are available on request from the corresponding author. The raw sequence files were deposited to the National Center for Biotechnology Information Sequence Read Archive with accession number SRP300881.

## Supplementary Material

Supplementary Table S1 Differentially accumulated metabolites.

Supplementary Table S2 KEGG analysis of differentially accumulated metabolites.

Supplementary Table S3 Differentially expressed genes.

Supplementary Table S4 GO and KEGG analyses of differentially expressed genes between LD and LW.

Supplementary Table S5 Primers used for qRT-PCR analysis.

Supplementary Table S6 Metabolite-gene pairs.
