# Supplementary material for: Metabolome and Transcriptome Analysis of Liver and Oocytes of Schizothorax o’connori Raised in Captivity
Source: Front Genet. 2021 Oct 8;12:677066. doi: 10.3389/fgene.2021.677066 (PMC8531413; doi:10.3389/fgene.2021.677066)
Supplement: Supplementary file 1 [file DataSheet1.ZIP › Supplementary_Material.docx]

Supplementary Material

# Supplementary Figures


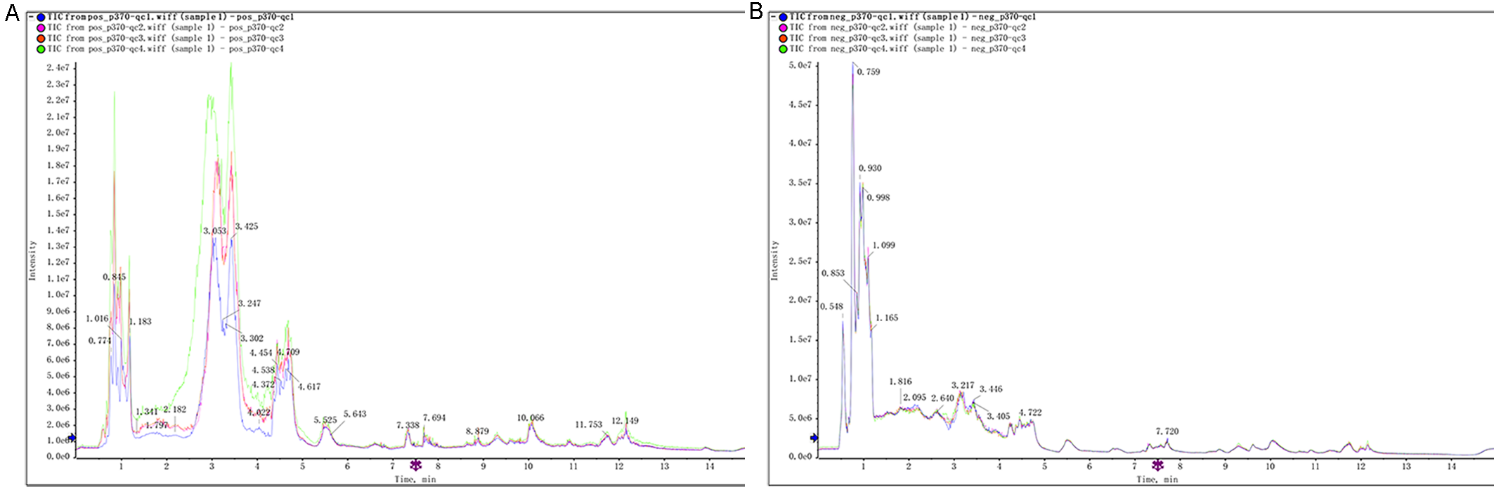


**Supplementary Figure 1.** Quality control sample of the overlay of all total ion current (TIC) chromatograms obtained in the (A) positive ion mode (ESI+) and (B) negative mode (ESI−). The y-axis represents the intensity.


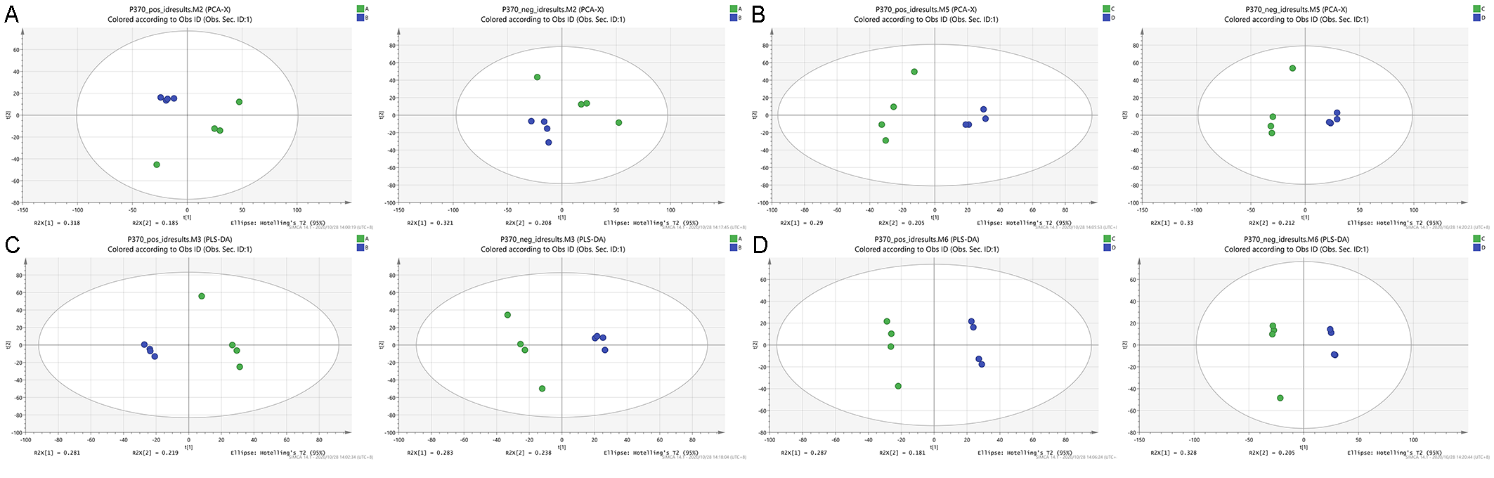


**Supplementary Figure 2**. Principal component analysis (A, B) and partial least squares-discriminant analysis (C, D) score plots, using the positive and negative ionization modes. Group A: oocyte from domestic *S. o’connori*. Group B: oocyte from wild *S. o’connori*. Group C. liver from domestic *S. o’connori*. Group D. liver from wild *S. o’connori*.


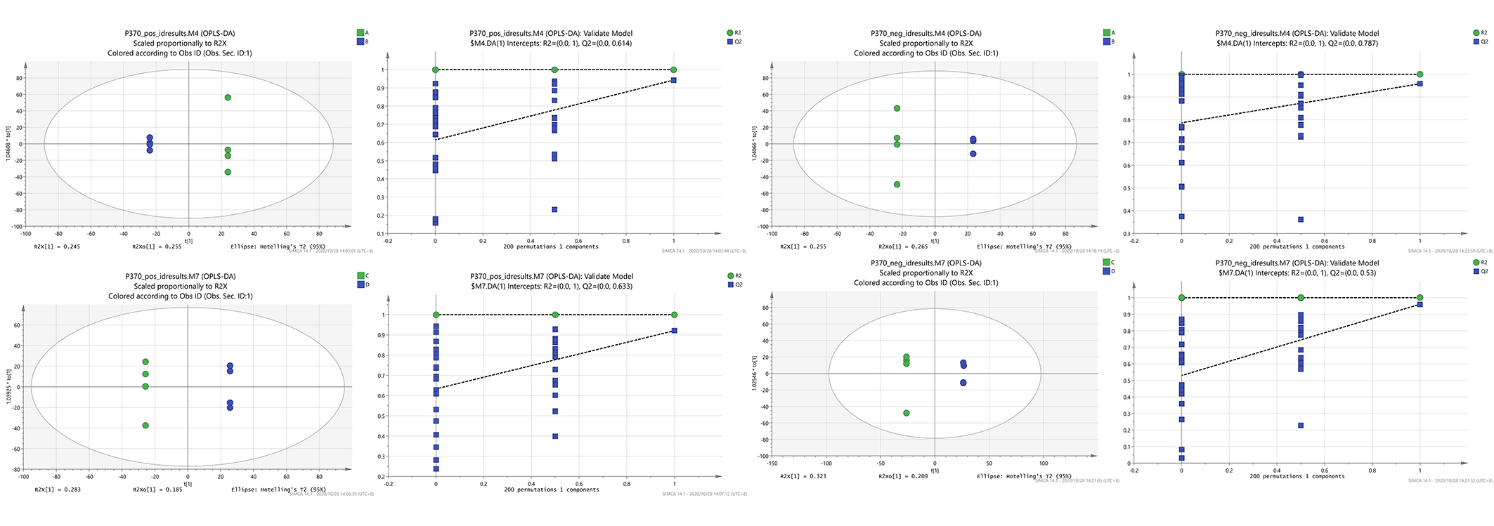


**Supplementary Figure 3**. Orthogonal partial least-squares-discriminant analysis (OPLS-DA) models using the positive and negative ionization modes. Group A: oocyte from domestic *S. o’connori*. Group B: oocyte from wild *S. o’connori*. Group C. liver from domestic *S. o’connori*. Group D. liver from wild *S. o’connori*.
