# Supplementary figures and images for: Metabolome and Transcriptome Analysis of Liver and Oocytes of Schizothorax o’connori Raised in Captivity
Source: Front Genet. 2021 Oct 8;12:677066. doi: 10.3389/fgene.2021.677066 (PMC8531413; doi:10.3389/fgene.2021.677066)

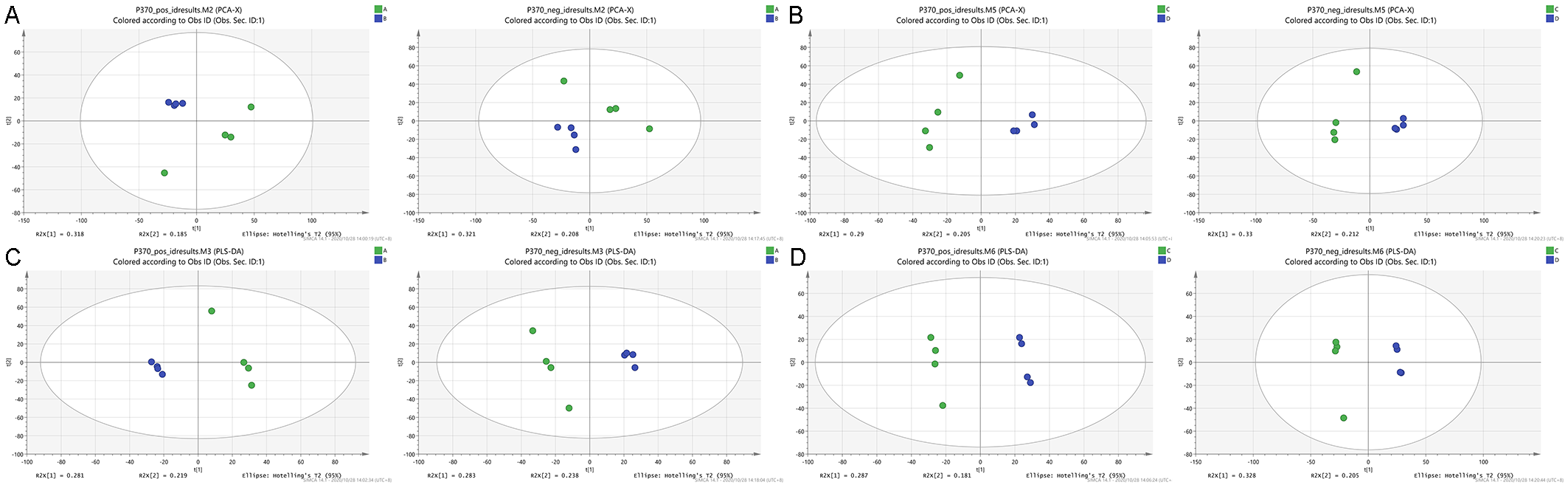

Supplement: Supplementary file 1 [file DataSheet1.ZIP › FIG S2.tif]

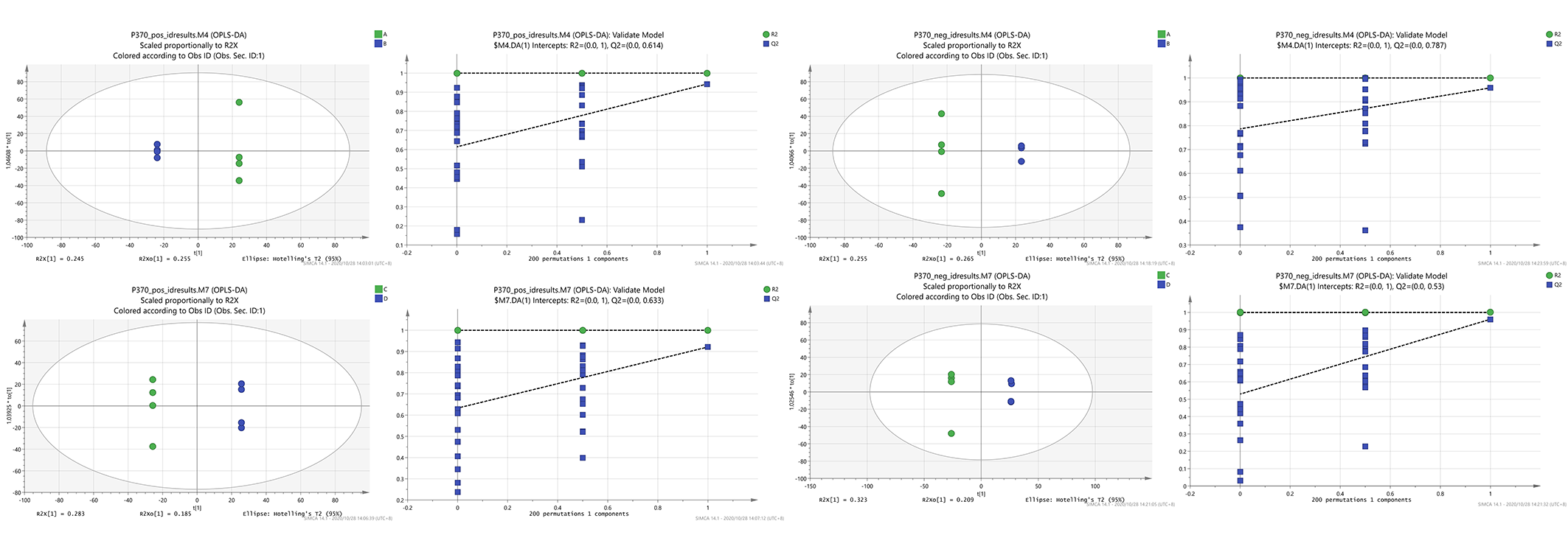

Supplement: Supplementary file 1 [file DataSheet1.ZIP › FIG S3.tif]

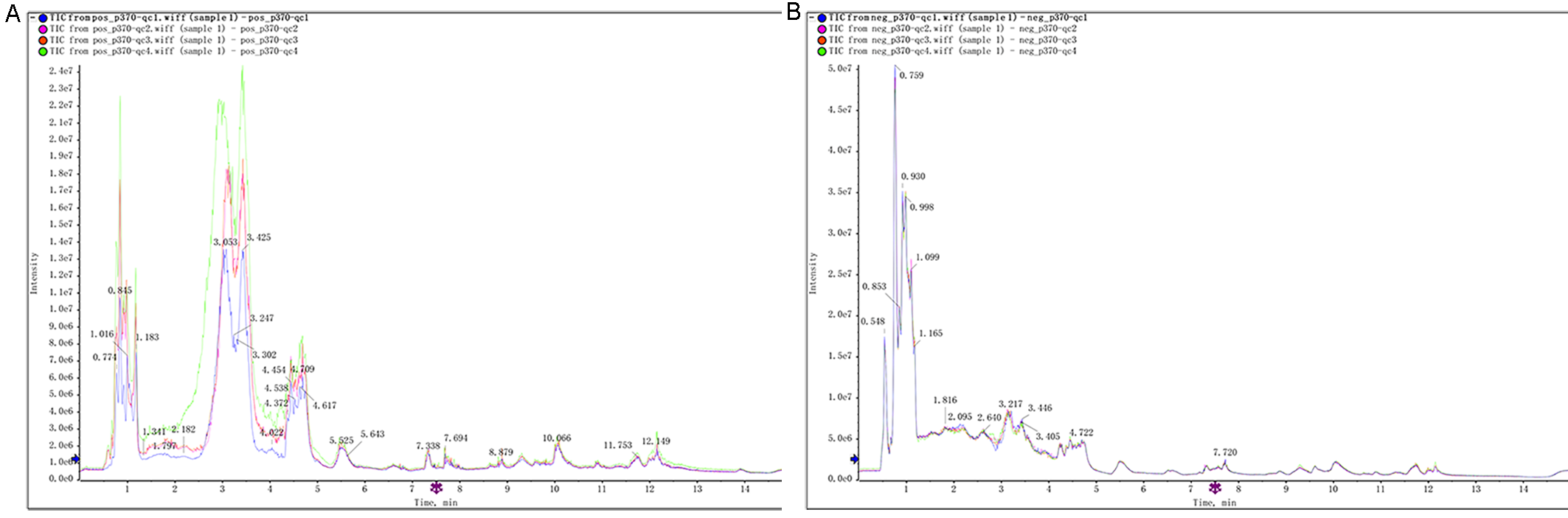

Supplement: Supplementary file 1 [file DataSheet1.ZIP › FIG S1.tif]
